# Postprandial dynamics of sensory receptor and neural mechanisms underlying feeding regulation in mule ducks

**DOI:** 10.1016/j.psj.2026.107070

**Published:** 2026-05-02

**Authors:** Marie Lasserre, Yan Fericelli, Cécile Heraud, Laura-Lou Zwick, Karel Sasso, Sandra Biasutti, Anne Surget, Marianne Houssier, Stéphane Panserat, Jérôme Roy, Karine Gontier

**Affiliations:** a Université de Pau et des Pays de l’Adour, INRAE, NUMEA, Mont-de-Marsan, France; b Université de Pau et des Pays de l'Adour, INRAE, NUMEA, Saint-Pée-sur-Nivelle, France

**Keywords:** Mule duck, Taste receptors, Nutrient sensing, Post-prandial kinetics, Feed intake regulation

## Abstract

The gustatory system plays a central role in nutrient detection and feeding regulation in birds, yet the underlying molecular mechanisms in ducks remain poorly characterized. Here, we provide the first integrated analysis of nutrient detection pathways, from peripheral oral sensing to central brain integration, in mule ducks at 4 weeks of age. Nutritional status was confirmed via plasma free fatty acids and triglycerides. We quantified taste receptors mediating sweet (Taste Receptor type 1 member 1 and 3), bitter (Taste Receptor type 2 member 9 and 40), sour (Acid-Sensing Ion Channels), umami (Metabotropic Glutamate Receptors 3 and 4), and lipid (G Protein-coupled Receptor 119 and family C group 6 member A) perception, together with key calcium-signaling components involved in gustatory transduction (Stromal Interaction Molecule 1 and Calcium release-activated calcium modulator 2), across the tongue, palate, and oral floor mucosa. Serotonin (5-hydroxytryptamine, **5-HT**) dynamics in these tissues were assessed as a peripheral neural readout. Feeding induced rapid, tissue-specific modulation of taste receptor and calcium-signaling gene expression, with the strongest effects consistently observed in the oral floor mucosa, likely reflecting species-specific oral anatomy and ingestion behavior. Postprandial feeding increased 5-HT levels in gustatory tissues. In contrast, hypothalamic neuropeptide expression remained largely unchanged, whereas central dopaminergic and serotonergic pathways exhibited feeding-dependent metabolic changes. Together, these findings reveal a dynamic peripheral gustatory system capable of detecting multiple nutrient classes and triggering rapid central neurochemical responses in ducks. This study provides a framework for understanding how oral nutrient detection interfaces with central feeding circuits and establishes a reference for developmental and nutritional studies targeting gustatory and neurochemical pathways in avian species.

## Introduction

Foie gras has been recognized as part of France’s cultural and gastronomic heritage since 2006 (Loi n°2006–11 du 5 janvier, 2006 reconnaissant le foie gras comme partie du patrimoine culturel et gastronomique de la France). According to a CIFOG/CSA survey carried out from November 27 to December 4, 2020, this high-quality product is regularly consumed by a large majority of the French population, and the foie gras duck and goose industry supports more than 100,000 direct and indirect jobs (Comité Interprofessionnel des Palmipèdes à Foie Gras (CIFOG) / CSA, 2020). However, foie gras production still depends on force-feeding. This practice, along with the housing conditions imposed during the force-feeding period (10 days of confinement), has come under increasing societal scrutiny from an animal-welfare perspective, highlighting the need for improvements. In this context, since 1999, the European Union has prohibited the establishment of foie gras production in countries without a historical tradition and has encouraged historically producing countries to explore alternatives to force-feeding (Council of Europe, 1999).

Foie gras production relies on hepatic steatosis, largely driven by excessive nutrient intake, particularly carbohydrates (Annabelle et al., 2017, 2018). Among the factors influencing liver fattening, feeding behavior, especially hyperphagia, plays a central role, making it a key target for strategies aiming to induce voluntary liver fattening.

In this context, several studies have investigated the possibility of inducing hepatic steatosis without force-feeding. In Greylag geese (*Anser anser*), this species can develop spontaneous hyperphagia accompanied by hepatic steatosis when exposed to specific photoperiod conditions and *ad libitum* corn feeding following a period of feed restriction (Guy et al., 2013; Fernandez et al., 2016). Spontaneous hyperphagia leads to a marked increase in liver weight and lipid content; however, the resulting livers remain considerably lighter and differ in lipid composition and sensory properties from those obtained through force-feeding (Fernandez et al., 2019). The process is lengthy (over 30 weeks) and associated with substantial variability in liver weight (coefficient of variation: 45 %). In addition, spontaneous fattening presents major drawbacks, including impaired sexual maturation, protein deficiencies resulting in reduced muscle growth, and increased mortality at the end of the hyperphagic period (Knudsen et al., 2025). Despite these limitations, these studies clearly highlight the central role of hyperphagia in the natural induction of hepatic steatosis, with increased feed intake emerging as the primary driver, along with enhanced lipogenesis and reduced hepatic fatty acid oxidation.

However, information on this phenomenon in ducks, the species predominantly used for foie gras production (FranceAgriMer, 2024) is very limited. This strategy has proven ineffective in ducks, which self-regulate feed intake very quickly (Zwick et al., 2025). It is therefore essential to understand how this species regulates its feed intake before attempting to induce voluntary hyperphagia.

In birds, as in mammals, the regulation of food intake relies heavily on sensory inputs, with the gustatory system playing a central role in detecting nutrients and guiding feeding behavior (Denbow, D. M.; Cogan, T., 2022a). Unlike in mammals, the avian tongue is not the main organ for taste perception: its epithelium is often keratinized and contains few taste buds (Erdoğan and Iwasaki, 2014; Niknafs et al., 2023). Most taste buds are instead located on the soft palate and the mandibular epithelium beneath the tongue (Marzban Abbasabadi and Sayrafi, 2018) and contain specialized sensory cells capable of detecting the primary taste modalities: bitter, sour, salty, sweet, umami, and fat (Niknafs et al., 2023). Nutrients such as glucose, amino acids, fatty acids, and minerals interact with gustatory receptors, triggering intracellular signaling pathways that induce neurotransmitter release. These signals are transmitted via cranial nerves, including the facial (VII), glossopharyngeal (IX), and vagus (X) nerves to the gustatory nuclei in the brainstem, and then relayed to the hypothalamus, where they modulate orexigenic (**AgRP**, Agouti-Related Peptide / **NPY,** Neuropeptide Y) and anorexigenic (**POMC,** Pro-opiomelanocortin / **CART,** Cocaine- and Amphetamine-Regulated Transcript) neurons involved in feeding control (Denbow, D. M.; Cogan, T., 2022b).

Beyond this sensory pathway, central neurotransmitters play a pivotal role in shaping feeding behavior. Serotonin (5-hydroxytryptamine, **5-HT**) is one of the best-characterized anorexigenic neurotransmitters: its activation in the hypothalamus enhances POMC neuron activity and inhibits AgRP/NPY neurons, ultimately reducing food intake. This mechanism is well established in mammals, and avian studies similarly show that stimulation of the serotonergic system decreases feeding motivation in chickens (Zhang et al., 2017). In parallel, dopamine participates in the hedonic and motivational dimension of feeding through mesolimbic reward circuits, reinforcing the consumption of palatable or energy-dense foods (Volkow et al., 2011). Collectively, these taste-dependent and neurotransmitter-mediated pathways integrate metabolic and reward-related signals to regulate feeding.

Chickens possess well-developed gustatory systems that allow them to detect and respond to a variety of nutrient stimuli, thereby modulating feed intake. They are sensitive to bitter compounds, amino acids, salty and sour stimuli, and fatty acids, while showing relatively low sensitivity to sugars (Liu et al., 2018; Yoshida et al., 2022). Amino acids such as glutamic acid, L-alanine, and L‑serine are detected through **T1R1**/**T1R3** receptors (Taste receptors type 1 member 1 and 3), and their presence in the diet has been shown to influence feed consumption (Moran and Stilborn, 1996; Yoshida et al., 2024). Lipid detection is also important, as chickens exhibit a preference for dietary fats, mediated by long-chain fatty acids via the **GPR120** receptor (G protein-coupled receptor 120) (Kawabata et al., 2021). Altogether, these findings highlight the diversity and complexity of gustatory mechanisms in chickens, their capacity to discriminate among nutrient classes, and the impact of these mechanisms on feeding preferences and poultry nutrition.

By contrast, no equivalent information is currently available regarding the identification, detection, or regulation of sensory systems involved in nutrient perception in ducks. Establishing a comprehensive overview of nutrient-detection pathways including gustatory receptors and their signaling cascades, as well as the associated central regulation via orexigenic and anorexigenic neuropeptides remains an unmet need for understanding feeding behavior in ducks.

The objective of this study is to characterize the nutrient detection pathways in the mule duck, from the oral cavity to central integration in the brain, in order to better understand the mechanisms regulating food intake. Specifically, we aim to identify gustatory receptors and their associated signaling pathways, as well as central responses mediated by orexigenic and anorexigenic neuropeptides and hormones, including dopamine (**DA**) and serotonin (5-HT), by comparing post-prandial and fasted individuals. This approach will provide unprecedented insights into the sensory and neurochemical mechanisms underlying feeding behavior in ducks, a species for which little information currently exists. Ultimately, these findings could guide the development of feeding strategies that stimulate voluntary hyperphagia, offering potential alternatives to traditional force-feeding practices.

## Materials and methods

### Animals and experimental procedures

All experimental procedures were conducted in accordance with French national guidelines for animal care in research at the certified Experimental Station for Waterfowl Breeding (INRAE 0089-AVIPOLE, Artiguères, France; accreditation number B40-037-1) and were evaluated and approved by the institutional Animal Welfare Body (SBEA). Male mule ducks (*n* = 180; genetic line MMG AS × PKL) were provided by MULOR (Carcarès-Sainte-Croix, France) at one day of age. Ducks were reared under standard light and temperature conditions and fed *ad libitum* from hatching to 4 weeks of age with a starter diet (PAG1301; 17.5 % crude protein, 2.85 % crude fat; 43.52 % starch; 2,800 kcal/kg metabolizable energy) (Table 1). This 4-week stage was selected as a key physiological window because the avian gustatory system was functionally mature (Gentle, 1972; Berkhoudt, 1992) and it precedes metabolic transitions and intensive fat deposition typical of later development (Chartrin et al., 2007; Bonnefont et al., 2019), allowing for the characterization of transcriptional responsiveness during a period of high growth.

**Table 1:** Ingredients and composition of the experimental starter diet (PAG1301, Nutricia, route de Saint Sever, Haut Mauco, BP27, France).

Commercial Diet PAG 1301		
Category	Constituent / Additive	Quantity
Analytical Constituents	Dry Matter (DM)	92 %
	Crude Protein	19.08 % DM
	Crude Fat	2.85 % DM
	Crude Ash	5.94 % DM
	Starch	43.52 % DM
	Energy (kJ.g^−1^)	18.48
Essential amino acids and minerals	Lysine	0.95 %
	Methionine	0.30 %
	Calcium	1.084 %
	Phosphorus	0.546 %
	Sodium	0.15 %
Vitamins	Vitamin D3 (3a671)	3000 IU/KG
	Vitamin A (3a672a)	7000 IU/KG
Amino Acids	Hydroxyl Analog of Methionine	0.11 %
Digestibility Enhancers	Endo-1,4-Beta-xylanase (EC 3.2.1.8)	150 FXU/KG
	4a19 6. Phytase (EC 3.1.3.26)	1000 FTU/KG
Trace Elements	Copper (3b4O5 sulfate II pentahydrate)	15 MG/KG
	Iron (3b103 sulfate (II) monohydrate)	24 MG/KG
	Manganese (3b502 oxide)	50 MG/KG
	Iodine (3b201 potassium iodide)	1.20 MG/KG
	Zinc (3b605 monohydrate sulfate)	35 MG/KG
	Selenium (sodium selenite 3b801)	0.20 MG/KG
Main Ingredients	Wheat	-
	Southwest Corn (Class A)	-
	Sunflower meal (feed extraction)	-
	Barley	-
	Wheat Bran	-
	Calcium Carbonate	-
	Sodium Chloride	-

At 4 weeks of age, ducks were randomly assigned to two experimental groups (*n* = 90 per group) using simple randomization: a Fed group, which received a meal, and a Fasted group, which did not receive feed prior to measurements. Before the kinetic study, all ducks were fasted overnight starting at 8:00 PM on the previous day, resulting in a 12 h fasting period at T0. Samples were collected at T0 (fasted baseline). In the Fed group, feeders were opened simultaneously in all boxes for 10 min, allowing ducks to consume the standardized meal. Feed intake was recorded for each box during this period. After 10 min, feeders were closed and kinetic measurements began. Measurements were performed at 20 min, 2 h, 6 h, and 12 h after meal administration in the Fed group. The same time points were applied to the Fasted group to allow comparison of fasting kinetics. To minimize handling-related stress, ducks were placed in separate boxes corresponding to each sampling time point, with two boxes per group to avoid potential box effects. At each time point, animals were weighed before sampling. Feed consumption was calculated as the difference between feed provided and feed remaining after the feeding period, expressed per animal by dividing by the number of ducks with access to the feeder.

Ducks (*n* = 10 per group per time point) were slaughtered by bleeding after electronarcosis. Blood was collected during bleed-out into EDTA tubes, and plasma was separated by centrifugation (2,000 g, 10 min, 4°C) and stored at −20°C. After dissection, the tongue, palate, mandible, and whole brain were collected at the same time points, rapidly frozen in liquid nitrogen, and stored at −80°C. Whole brains were systematically collected to enable both hypothalamus microdissection for **RT-qPCR** (Reverse transcription - Polymerase chain reaction) analyses and whole-brain homogenization for **UHPLC-FL** (Ultra-High Performance Liquid Chromatography with Fluorescence detection) quantification.

### Biochemical assays

Plasma triglycerides (**TG**) and non-esterified fatty acids (**NEFA**) were quantified using enzymatic colorimetric kits: the GPO-POD kit (Sobioda, France) for TG, and the NEFA HR2 kit (Wako, Richmond, VA, USA; product reference: W1W436-91995) for NEFA.

### RNA extraction and quantitative real-time PCR (RT-qPCR)

Total RNA was isolated from frozen tissues (tongue, palate, oral floor mucosa, and hypothalamus) using the TRIzol method (Invitrogen, Carlsbad, CA, USA; Thermo Fisher Scientific, 12034977) according to the manufacturer’s instructions. RNA integrity was verified by electrophoresis on a 1 % agarose gel, and concentrations were measured spectrophotometrically at 260 nm using a Biotek EPOCH 2 microplate reader (Take3 plate). All samples were adjusted to 500 ng/µl prior to reverse transcription. Reverse transcription was performed in duplicate using 1 µg of RNA with SuperScript III Reverse Transcriptase (Invitrogen; 18080044), Random Primers (Promega, Madison, WI, USA; C1181), and PCR nucleotide mix (Promega, C1145). Negative controls (no-RT and no-template) were included to verify the absence of contamination. Gene expression was quantified by real-time PCR using PerfeCTa SYBR Green FastMix (VWR, Radnor, PA, USA; 733-1379) on a CFX384 system (Bio-Rad). Primers for genes described for the first time in ducks were designed in-house; their specificity was confirmed by Sanger sequencing (GeneWiz) and further verified by melting-curve analysis, and only primers with an efficiency ≥85 % were retained for analysis. Gene-specific primers were used at a final concentration of 400 nM. Full details of the primer sequences, are summarized in Table 2. Each reaction contained cDNA, SYBR Green mix, and nuclease-free water, and was run in duplicate. Ct values from duplicates were averaged, and samples with differences >1 Ct were excluded. Relative expression was calculated using the 2^-ΔΔCt method, with ΔCt defined as the difference between the Ct of the target gene and the geometric mean of the reference genes (*EF1* and *ACTIN B* or *EF1* and *GAPDH*, depending on the tissue), and all samples were normalized to the corresponding ΔCt at T0. Raw Ct values are provided in Supplementary data.

**Table 2:** Nucleotide sequences of the PCR primers used to evaluate the mRNA abundance of transcripts by real-time quantitative PCR (5′−3′ sens).

Gene family	Gene	Forward (5′−3′)	Reverse
Taste receptor	*t1r1*	AGGTCAATGTCACCGTCGTC	ACATCCCAATCACAGAGCCG
	*t1r3*	TCTTGATCCCACAGGTCAGCTA	CCAACGTTGCGCAAGAAGAG
	*t2r9*	CACCTGCGCTGCTCTAAAAAG	TGCTCAGCATAGGATGGCAG
	*t2r40*	ATCCTAGCCAAGAGGCCCAT	CGAAATTTGGGGTTGCTCCA
Acid sensing ion channel	*asic4*	AGTCCATCTGCTCTCCCAAC	TTCTTGTTGTACTTGCGGGC
	*asic1-2*	TAGCCACAGGGGGAGGTATC	ATAGCCTCGGCGGTACCTTG
Glutamate metabotropic receptor	*grm3*	AGGAGTCATAGGCGGTTCCT	AACCGTTGACACGTAGGTCC
	*grm4*	TCGCAGTCGGCAGAAAAGAT	TCCGGGTGGAAGAGGATGAT
G protein receptor	*gpRc6A*	CTTTTTGGCTCAGGGCACAC	TTGACTTCTCTGGACGGCAC
	*gpr119*	AACCCCCTGCTCTACTCCTG	TGAGTTCATGGTTGTGGCAGA
Stromal interaction molecule	*stim1*	CTCCCATGTCCATACAATCTCC	AAAAAGCCAGGCTGCATATTGTA
Calcium release-activated calcium modulator	*orai2*	GGAAGTGGGTGGTCACGAAA	AGCCCTATTCTTCCTGCCGT
Orexigenic neuropeptides	*npy*	ATCACCAGGCAGAGGTATGGA	CACCACATCGAAGGGTCTTCA
	*agrp*	CTGGAACCGCAGGCATCAT	GTTGAAGAAGCGGCAGTAGC
Anorexigenic neuropeptides	*cartp*	ACTCCTTCCTCCTCAAGTGC	GGACAGGGAAGAGAACAGCT
	*pomc*	GAGAACGGGGTTTTGGCGTG	GCCGAATTTGTTCCAGCGAA
Melanocortin 4 receptor	*mc4r*	AATGCAAAGGGCCACTCCTC	GTACATGGGCGAATGGAGGT

### Indolamine and catecholamine measurement using UHPLC-FL

Indolamines and catecholamines were quantified in tongue, palate, oral floor mucosa, and brain tissues (*n* = 10) using UHPLC (ACQUITY H—Class PLUS, Waters, Milford, MA, USA) equipped with a thermostatted autosampler (4°C) and a fluorescence detector (ACQUITY FLR, Waters). All reagents and standards were purchased from Merck KGaA (Darmstadt, Germany), and organic solvents were gradient MS grade (ADL & Prochilab, Lormont, France). Tissues were homogenized using a Precellys® 24 homogenizer (Bertin Technologies, Montigny-le-Bretonneux, France) in 20 mM phosphate buffer with 1 mM EDTA (pH 6.5 ± 0.05) at a 1:5 (m/v) ratio. After centrifugation (14,000 g, 20 min, 4°C), supernatants were deproteinized with an equal volume of 10 % metaphosphoric acid, centrifuged again (14,000 g, 5 min, 4°C), and filtered through a 0.22 µm PVDF membrane. Samples were stored at −20°C until analysis. Chromatographic separation was performed on a Luna® PFP(2) column (150 × 4.6 mm, 3 µm, Phenomenex) at 30°C. Injection volume was 10 µl, flow rate was 0.4 ml/min. A quaternary solvent system was used: (A) 10 mM phosphate buffer (pH 4.3 ± 0.05), (B) methanol, and (C) ultrapure water. The 25 min following linear gradient elution was used: 0 min: 85 % A, 15 % B; 6 min: 80 % A, 20 % B; 7.5 min: 70 % A, 30 % B; 9 min: 60 % A, 40 % B; 19.2 min: 85 % A, 15 % B. The mobile phase was filtered through 0.2 µm in-line membrane filters. Linear gradient elution was applied (0-30 min). Fluorescence detection was performed at two wavelength sets: 285 nm (excitation) / 355 nm (emission) for indoles (5-hydroxytryptamine serotonin: 5-HT, 5-hydroxyindolacetic acid: **5-HIAA**) and 280 nm (excitation) / 330 nm (emission) for catecholamines (3,4-dihydroxyphénylalanine: **l-DOPA**, homovanillic acid: **HVA**). Metabolites were identified by comparing retention times to standards and quantified by integrating peak areas against calibration curves (R² > 0.995, linear range 0.05-55 pmol per injection). Limits of detection (LLOD) and quantification (LLOQ) were determined based on signal-to-noise ratios of 3 and 10, respectively, and are reported in Supplementary Table 1. Concentrations were normalized to total protein content measured by the bicinchoninic acid method (#BCA1-1KT, Sigma-Aldrich). The method was adapted from Heraud et al. (2022).

### Statistical analysis

Statistical analyses were performed using R software (version 3.6.2) with RStudio. Data normality and homoscedasticity were assessed for each variable using Shapiro-Wilk and Levene tests, respectively. When assumptions were met (*p* > 0.05), a two-way ANOVA was conducted to evaluate the effects of nutritional status (Fed vs. Fasted), time (T0, 20 min, 2 h, 6 h, 12 h), and their interaction, followed by Tukey’s post hoc test when significant effects were detected. For variables violating assumptions, data were log-transformed and re-analyzed. If assumptions remained unmet or sample sizes were too small (*n* < 6), a non-parametric Scheirer-Ray-Hare test was applied, followed by Dunn’s post hoc test. Results were presented as means ± SEM, and statistical significance was set at *P* < 0.05.

## Results

We first examined the effects of feeding on peripheral gustatory tissues and central neurochemical pathways by analyzing metabolic status, taste receptor expression, and neuropeptide and monoamine profiles in mule ducks.

### Plasma triglycerides and free fatty acids (Fig. 1)

Plasma free fatty acids (**FFA**) and triglycerides (TG) were measured to assess the nutritional status of the animals. In the Fed group, FFA decreased after feeding, reaching a minimum around 2 h (0.11 g/L) and remained low until a slight rise at 12 h (0.18 g/L), while TG rapidly increased to a peak at 20 min (1.1 g/L) and stayed elevated for several hours. In contrast, FFA levels in the Unfed group remained higher than in Fed animals at 2 h (0.26 *vs*. 0.11 g/L), whereas TG stayed low throughout the experiment (≈0.5 g/L). Statistical analysis revealed a significant interaction between time and feeding status, with both factors exerting significant effects (*P* < 0.05). To further characterize lipid dynamics, a first-order kinetic analysis was applied to the TG elimination phase (20 min to 12 h). The semi-logarithmic transformation (ln) revealed a clear clearance trend in both groups, with the Unfed group exhibiting a twofold higher elimination rate constant (*k* = 0.0526 h^−1^) compared to the Fed group (*k* = 0.0255 h^−1^), suggesting a faster turnover of circulating triglycerides in the fasted state.

**Fig. 1:**
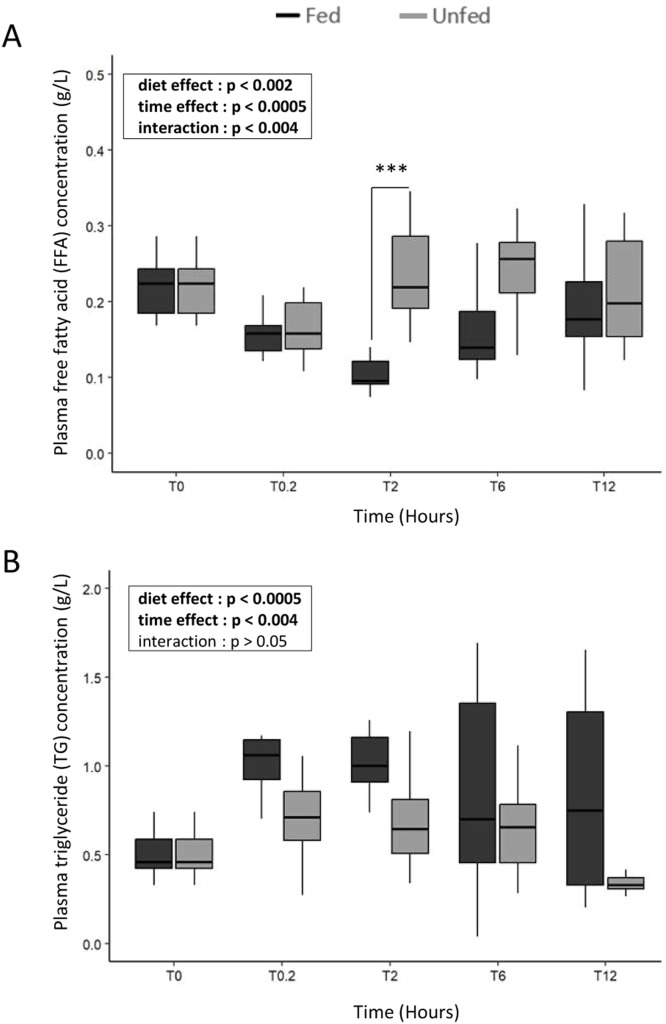
**Plasma concentrations of FFA (A) and TG (B) in fed and unfed mule ducks.** Data are shown as boxplots (median and IQR). Statistical analyses were performed using two-way ANOVA or non-parametric tests (Scheirer-Ray-Hare, Kruskal-Wallis or Mann-Whitney) depending on data distribution. Significant differences are indicated by asterisks (* *P* < 0.05, ** *P* < 0.01, *** *P* < 0.001; *n* = 10).

### Taste receptor gene expression (Fig. 2, Fig. 3, Fig. 4, Fig. 5, Fig. 6)

In the tongue, only a subset of sweet taste receptor genes was reliably detected. *T1R1* transcripts were present, whereas *T1R3* remained below the detection threshold (Ct > 35). *T1R1* expression showed a significant diet × time interaction, with higher levels in unfed ducks at 2 h. Among other receptor families, bitter receptor ***T2R9*** (Taste receptor type 2 member 9) and umami receptor ***GRM4*** (Metabotropic glutamate receptor 4) exhibited significant time-dependent variations, whereas ***T2R40*** (Taste receptor type 2 member 40), ***GRM3*** (Metabotropic glutamate receptor 3), and ***GPR119*** (G protein-coupled receptor 119) remained stable. In contrast, ***GPRC6A*** (G protein-coupled receptor family C group 6 member A) expression decreased over time (*P* < 0.05), and ***ASIC1-2*** (Acid-Sensing Ion Channel 1-2) also showed a significant time-dependent variation, while ***ASIC4*** (Acid-Sensing Ion Channel 4) expression remained unchanged.

**Fig. 2:**
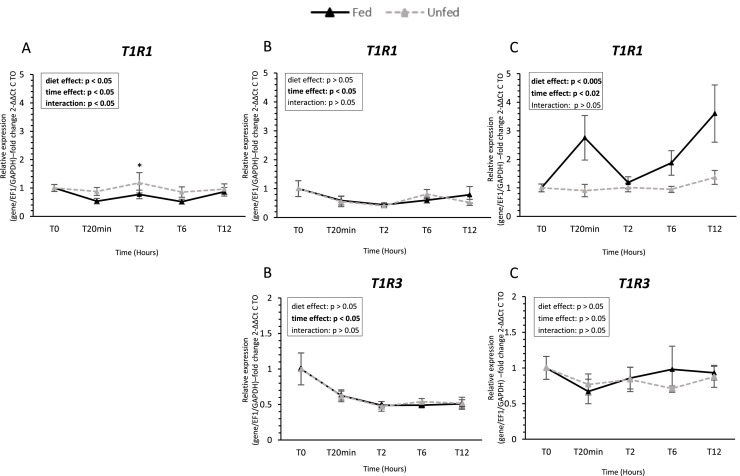
S**weet taste receptor gene expression in oral tissues of fed and unfed mule ducks.** Relative mRNA expression of sweet taste receptor subunits (*T1R1* and *T1R3,* Taste receptors type 1 member 1 and 3) in the tongue (A), palate (B), and oral floor mucosa (C) of fed and unfed mule ducks measured at T0, 20 min, 2 h, 6 h, and 12 h. Data are presented as median (IQR). Fold changes were calculated using 2^−ΔΔCt^ method relative to T0. Statistical analyses were performed using two-way ANOVA or non-parametric tests (Scheirer-Ray-Hare, Kruskal-Wallis or Mann-Whitney) depending on data distribution. Significant differences are indicated by asterisks (* *P* < 0.05, ** *P* < 0.01, *** *P* < 0.001; *n* = 10).

**Fig. 3:**
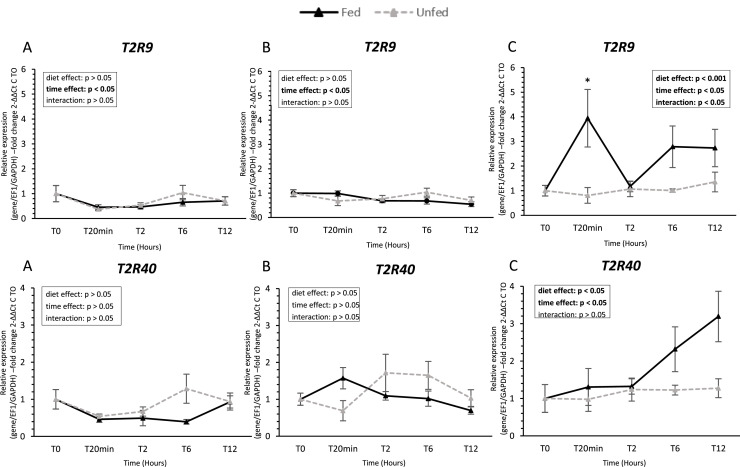
**Bitter taste receptor gene expression in oral tissues of fed and unfed mule ducks.** Relative mRNA expression of bitter taste receptor subunits (*T2R9* and *T2R40*, Taste receptors type 2 member 9 and 40) in the tongue (A), palate (B), and oral floor mucosa (C) of fed and unfed mule ducks measured at T0, 20 min, 2 h, 6 h, and 12 h. Data are presented as median (IQR). Fold changes were calculated using 2^−ΔΔCt^ method relative to T0. Statistical analyses were performed using two-way ANOVA or non-parametric tests (Scheirer-Ray-Hare, Kruskal-Wallis or Mann-Whitney) depending on data distribution. Significant differences are indicated by asterisks (* *P* < 0.05, ** *P* < 0.01, *** *P* < 0.001; *n* = 10).

**Fig. 4:**
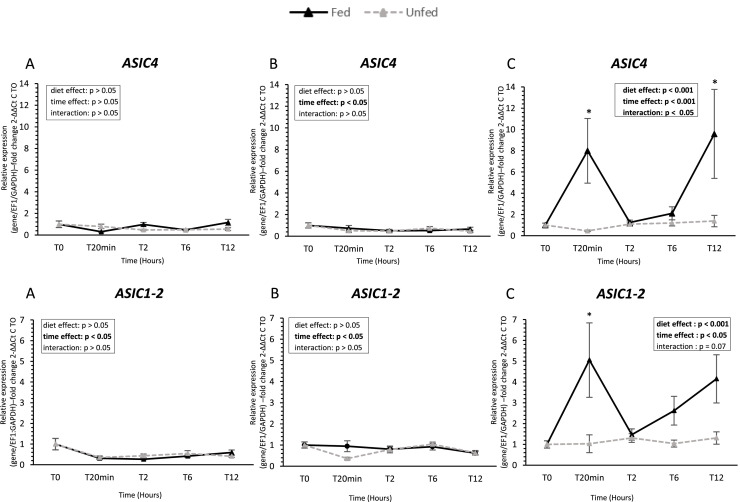
**Acid-sensing ion channel gene expression in oral tissues of fed and unfed mule ducks.** Relative mRNA expression of acid-sensing ion channels (*ASIC4 and ASIC1-2*, Acid-Sensing Ion Channels 1-2 and 4) in the tongue (A), palate (B), oral floor mucosa (C) of fed and unfed mule ducks measured at T0, 20 min, 2 h, 6 h, and 12 h. Data are presented as median (IQR). Fold changes were calculated using 2^−ΔΔCt^ method relative to T0. Statistical analyses were performed using two-way ANOVA or non-parametric tests (Scheirer-Ray-Hare, Kruskal-Wallis or Mann-Whitney) depending on data distribution. Significant differences are indicated by asterisks (* *P* < 0.05, ** *P* < 0.01, *** *P* < 0.001; *n* = 10).

**Fig. 5:**
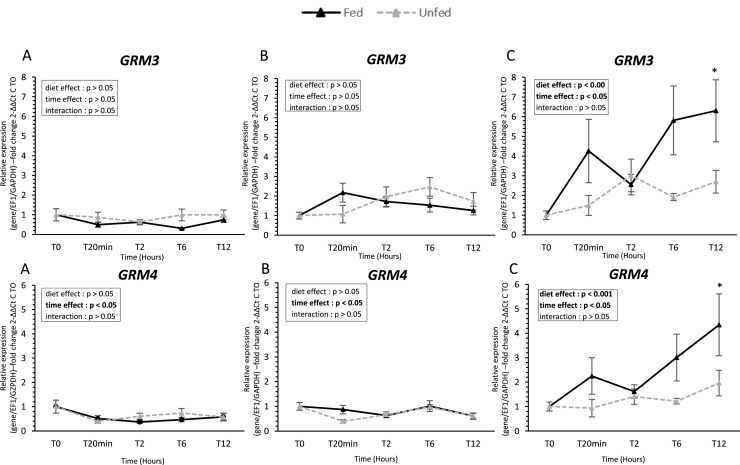
**Umami receptor gene expression in oral tissues of fed and unfed mule ducks.** Relative mRNA expression of umami receptors (*GRM3* and *GRM4*, Metabotropic glutamate receptors 3 and 4) in the tongue (A), palate (B), and oral floor mucosa (C) of fed and unfed mule ducks measured at T0, 20 min, 2 h, 6 h, and 12 h. Data are presented as median (IQR). Fold changes were calculated using 2^−ΔΔCt^ method relative to T0. Statistical analyses were performed using two-way ANOVA or non-parametric tests (Scheirer-Ray-Hare, Kruskal-Wallis, Mann-Whitney) depending on data distribution. Significant differences are indicated by asterisks (* *P* < 0.05, ** *P* < 0.01, *** *P* < 0.001; *n* = 10).

**Fig. 6:**
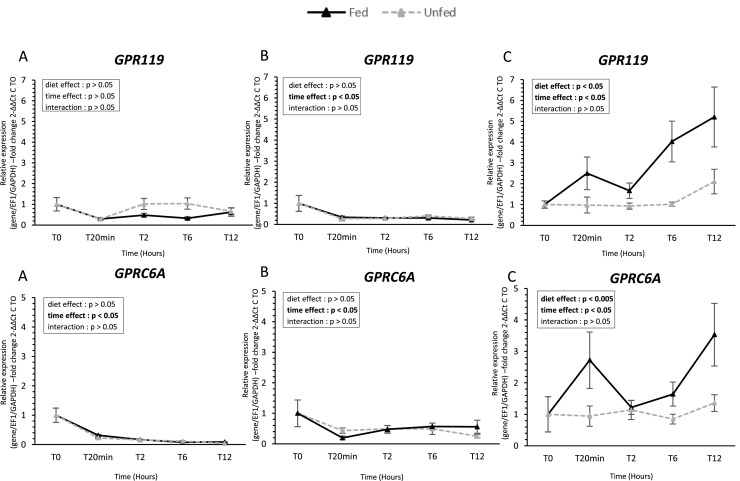
**Fatty acid receptor gene expression in oral tissues of fed and unfed mule ducks.** Relative mRNA expression of fatty acid receptors (*GPR119*, G protein-coupled receptor 119, and *GPRC6A*, G protein-coupled receptor family C group 6 member A) in the tongue (A), palate (B), and oral floor mucosa (C) of fed and unfed mule ducks measured at T0, 20 min, 2 h, 6 h, and 12 h. Data are presented as median (IQR). Fold changes were calculated using 2^−ΔΔCt^ method relative to T0. Statistical analyses were performed using two-way ANOVA or non-parametric tests (Scheirer-Ray-Hare, Kruskal-Wallis, Mann-Whitney) depending on data distribution. Significant differences are indicated by asterisks (* *P* < 0.05, ** *P* < 0.01, *** *P* < 0.001; *n* = 10).

In the palate, both sweet receptors (*T1R1* and *T1R3*) were detected and displayed significant time-dependent variations, with a marked decrease at 20 min post-feeding (*P* < 0.05). Bitter receptor *T2R9* and umami receptor *GRM4* also displayed significant temporal variations, whereas *T2R40* and *GRM3* expression remained stable regardless of time or diet group. Similarly, both *GPR119* and *GPRC6A* showed a significant decrease over time (*P* < 0.05), with no effect of diet. *ASIC1-2* and *ASIC4* were also affected by time, but no consistent diet effect was observed across receptors in this tissue.

In contrast, the oral floor mucosa exhibited the most pronounced response to feeding. *T1R1* expression increased significantly after feeding (*P* < 0.005), while *T1R3* remained unchanged. Both bitter receptors (*T2R9* and *T2R40*) were upregulated after feeding, with *T2R9* showing higher expression in fed ducks at 20 min post-feeding. Similarly, *ASIC1-2* and *ASIC4* were significantly higher in fed ducks, with early postprandial upregulation. Umami receptors *GRM3* and *GRM4* were also upregulated in fed ducks, showing significant effects of both time and diet (*P* < 0.05). Finally, lipid-related receptors *GPR119* and *GPRC6A* were consistently higher in fed ducks, with significant effects of feeding status and time (*P* < 0.05).

### Calcium-signaling pathway gene expression (Fig. 7)

***STIM1*** (Stromal Interaction Molecule 1) expression varied significantly over time in all three tissues (tongue, palate, and oral floor mucosa; *P* < 0.05). In contrast, ***ORAI2*** (Calcium release-activated calcium modulator 2) expression showed significant temporal variation only in the tongue.

**Fig. 7:**
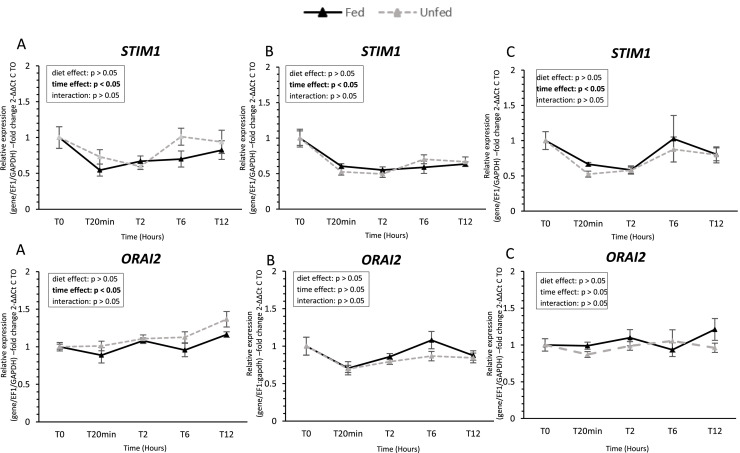
**Calcium-signaling gene expression in oral tissues of fed and unfed mule ducks.** Relative mRNA expression of calcium-signaling pathways genes (*STIM1*, Stromal Interaction Molecule 1, and *ORAI2*, Calcium release-activated calcium modulator 2) in the tongue (A), palate (B), and oral floor mucosa (C) of fed and unfed mule ducks measured at T0, 20 min, 2 h, 6 h, and 12 h. Data are presented as median (IQR). Fold changes were calculated using the 2^−ΔΔCt^ method relative to T0. Statistical analyses were performed using two-way ANOVA or non-parametric tests (Scheirer-Ray-Hare, Kruskal-Wallis or Mann-Whitney), depending on data distribution. Significant differences are indicated by asterisks (* *P* < 0.05, ** *P* < 0.01, *** *P* < 0.001; *n* = 10).

### Kinetics of indolamine metabolites (5-HT and 5-HIAA) (Fig. 8)

5-HT levels increased after feeding and were consistently higher in fed ducks, with significant diet × time interactions detected in all tissues (at 2 h in the tongue, 2 h and 6 h in the palate, and as early as 20 min in the oral floor mucosa). For 5-HIAA, no significant diet effect was detected in the tongue or palate; however, in the oral floor mucosa, levels were higher in unfed ducks at 20 min and 2 h post-feeding. Consequently, the 5-HIAA/5-HT ratio exhibited a diet × time interaction in the tongue and palate, with higher values in unfed ducks at 6 h, and as early as 20 min in the oral floor mucosa.

**Fig. 8:**
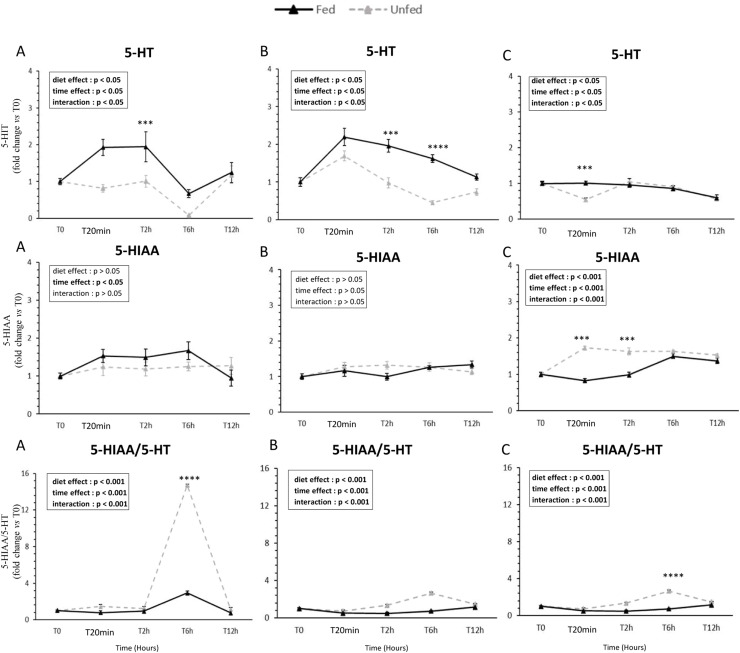
**Feeding effects on serotonin turnover in oral tissues of mule ducks.** Influence of feeding on serotonin turnover in the tongue (A), palate (B), and oral floor mucosa (C). Concentrations of indolamines (5-HT, 5-hydroxytryptamine, 5-HIAA, 5-hydroxyindolacetic acid) and turnover ratio (5-HIAA/5-HT) in fed and unfed mule ducks at T0, 20 min, 2 h, 6 h, and 12 h. Data are presented as means ± SEM; fold changes are expressed relative to T0. Statistical analyses were performed using two-way ANOVA or non-parametric tests (Scheirer-Ray-Hare, Kruskal-Wallis or Mann-Whitney) depending on data distribution. Significant differences are indicated by asterisks (* *P* < 0.05, ** *P* < 0.01, *** *P* < 0.001; *n* = 10).

### Neuropeptide and monoamine profiles in the brain (Figs. 9 and 10)

Among hypothalamic anorexigenic markers, *CARTP* expression decreased over time (*P* < 0.05) and ***MC4R*** (Melanocortin 4 receptor) increased (*P* < 0.05), while *POMC* remained stable; no neuropeptide showed a diet effect. Regarding brain indolamines, 5-HT levels increased in fed ducks, with a significant diet × time interaction at 2 h post-feeding. 5-HIAA, the main 5-HT metabolite, was reduced and significantly more pronounced in the fed group; consequently, the 5-HIAA/5-HT ratio decreased in the fed group (time and diet effects). In the catecholamine pathway, l-DOPA levels were higher in unfed ducks (*P* < 0.05), and HVA levels were reduced after feeding, leading to a postprandial decrease in the HVA/L-DOPA ratio in unfed ducks.

**Fig. 9:**
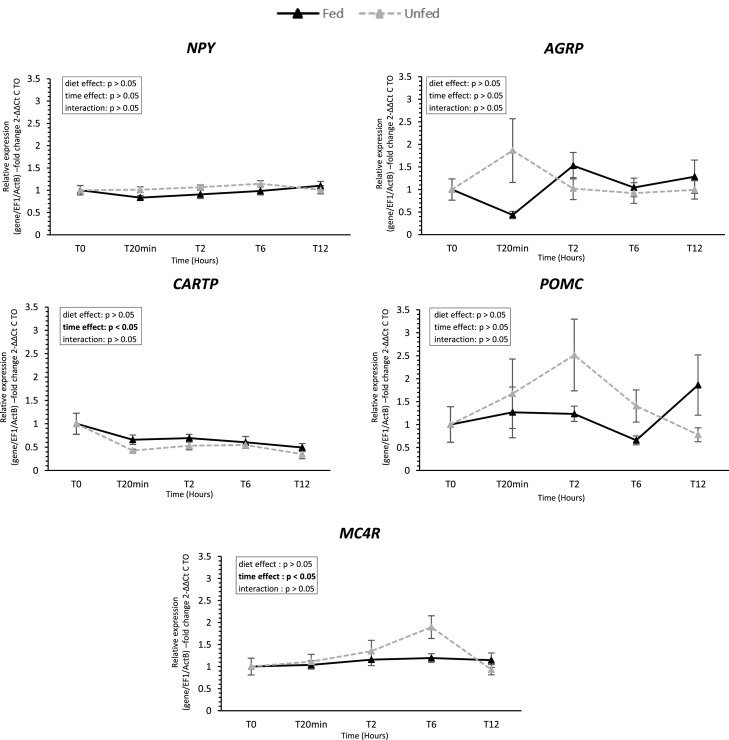
**Hypothalamic expression of feed intake regulation genes in fed and unfed mule ducks.** Relative mRNA expression of genes involved in feed intake regulation (*NPY*, Neuropeptide Y*, AGRP*, Agouti-Related Peptide*, CARTP*, Cocaine- and Amphetamine-Regulated Transcript Peptide*, POMC*, Pro-opiomelanocortin, and *MCR4*, Melanocortin-4 Receptor) in the hypothalamus of fed and unfed mule ducks measured at T0, 20 min, 2 h, 6 h, and 12 h. Data are presented as median (IQR). Fold changes were calculated using the 2^−ΔΔCt^ method relative to T0. Statistical analyses were performed using two-way ANOVA or non-parametric tests (Scheirer-Ray-Hare, Kruskal-Wallis or Mann-Whitney), depending on data distribution. Significant differences are indicated by asterisks (* *P* < 0.05, ** *P* < 0.01, *** *P* < 0.001; *n* = 10).

**Fig. 10:**
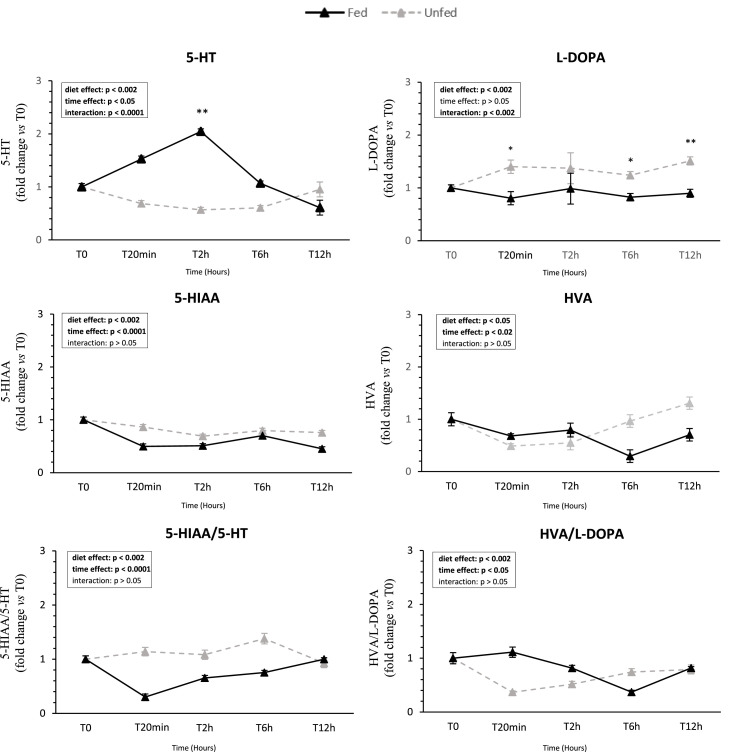
**Influence of feeding on serotonin and dopamine turnover in brain tissue**. Concentrations of indolamines (left: 5-HT, 5-hydroxytryptamine or serotonin and 5-HIAA, 5-hydroxyindolacetic acid) and turnover ratio (5-HIAA/5-HT) and catecholamines (right: l-DOPA or l-3,4-dihydroxyphenylalanine and HVA, Homovanillic acid) and turnover ratio (HVA/L-DOPA) in fed and unfed mule ducks at T0, 20 min, 2 h, 6 h and 12 h. Data are presented as means ± SEM; fold changes are expressed relative to T0. Statistical analyses were performed using two-way ANOVA or non-parametric tests (Scheirer-Ray-Hare, Kruskal-Wallis or Mann-Whitney), depending on data distribution. Significant differences are indicated by asterisks (*P < 0.05, ** *P* < 0.01, *** *P* < 0.001; *n* = 10).

## Discussion

The gustatory system plays a central role in early nutrient detection in birds (Denbow, D. M.; Cogan, T., 2022b). Beyond a few studies describing tongue anatomy, morpho-functional adaptations of the lingual mucosa to feeding strategies, and the distribution and cellular structure of taste buds in ducks (Stornelli et al., 2000; Skieresz-Szewczyk and Jackowiak, 2016; Marzban Abbasabadi and Sayrafi, 2018), the literature remains limited. Molecular mechanisms underlying flavor detection have been relatively well described in chickens, including the expression and function of key taste receptors and associated signaling pathways. In contrast, these pathways remain limitedly characterized in ducks.

In the present study, we present the first dynamic characterization of taste receptor expression, calcium-signaling pathways, and associated neurochemical mechanisms in mule ducks under fed versus fasted conditions. This approach allowed us to examine how this species can detect nutrients associated with different taste modalities, including sweet, bitter, sour, umami, and lipid, following a meal, as previously described in chickens (Liu et al., 2018; Yoshida et al., 2022).

Differences in circulating FFA and TG levels confirmed that fed and fasted ducks were in distinct nutritional states, consistent with reports showing that fasting enhances lipid mobilization and alters circulating metabolites in avian species (Liu et al., 2024). The first-order kinetic analysis further solidifies this distinction, revealing a twofold increase in the triglyceride elimination rate (*k*) in fasted ducks compared to fed ones. This accelerated clearance, characteristic of a negative energy balance, provides a reliable foundation to interpret subsequent postprandial molecular and neurochemical responses when studying peripheral and central feeding-related signals.

### Peripheral taste receptor responses

In the postprandial state, we evaluated the gene expression of key taste receptors involved in sweet, bitter, sour, umami, and lipid perception in fed ducks and compared it with that of fasted ducks across three oral tissues: the tongue, the palate, and the oral floor mucosa.

In mammals, sweet taste is mediated by the T1R2/T1R3 heterodimeric receptor, whereas umami stimuli are detected by T1R1/T1R3 receptor (Nelson et al., 2001). In birds, the T1R2 gene is generally absent, distinguishing them from mammals. In some species, such as hummingbirds, the ancestral T1R1/T1R3 receptor has been repurposed for sugar detection (Baldwin et al., 2014). Consistent with this evolutionary pattern, in mule ducks, *T1R1* and *T1R3* were expressed across all oral tissues studied, exhibiting clear feeding-dependent and tissue-specific modulation, particularly in the oral floor mucosa. These expression profiles could support the existence of a functional T1R1/T1R3 receptor complex in ducks, although its precise ligand specificity in ducks remains to be experimentally determined. Similar expression patterns have been reported in other avian species such as chicken, although cellular co-expression appears limited, reflecting species-specific variation in receptor distribution and potential ligand specificity (Moran and Stilborn, 1996; Yoshida et al., 2022, 2024). Altogether, these data suggest that carbohydrate detection via T1R1/T1R3 represents a conserved gustatory strategy in birds, with nuanced differences across species.

Bitter taste, mediated by T2R receptors, plays a crucial role in detecting toxic compounds such as plant alkaloids and insect defensive secretions. Comparative genomic analyses across bird indicate that the number of functional *TAS2R* genes varies widely among species and correlates with dietary toxin exposure (Li and Zhang, 2014; Wang and Zhao, 2015; Kawabata and Tabata, 2022). Ducks, as omnivorous birds consuming both plant material and invertebrates, are exposed to a range of potentially harmful compounds. In this ecological context, the expression and postprandial modulation of *T2R9* and *T2R40* observed in the oral floor mucosa of mule ducks align with predictions from comparative genomic studies, supporting the idea that bitter taste receptor regulation reflects dietary exposure to toxins.

ASIC channels, expressed throughout the oral cavity including the oral floor mucosa and palate, contribute to acid nociception and modulate taste sensitivity at lower pH thresholds (Jasti et al., 2007; Zhang et al., 2025). This modality is particularly relevant in duck production systems, where fermented or acidified diets are increasingly used to improve nutrient digestibility, modulate gut microbiota, and enhance growth performance (Hamid et al., 2018; Li et al., 2022). In the present study in mule ducks, *ASIC1-2* and *ASIC4* were expressed in all oral tissues studied, with higher postprandial modulation observed in the oral floor mucosa. These results support a role for acid sensing not only in detecting acidity but also in assessing food quality, including fermentation status, which may influence feeding behavior and digestive processes.

Umami taste, associated with amino acid detection, involves GRM3 and GRM4 in birds (Niknafs et al., 2023). In mule ducks, both *GRM3* and *GRM4* were expressed in all oral tissues studied, with pronounced postprandial modulation in the oral floor mucosa. Functional studies in chickens have shown that l-glutamate, especially in combination with nucleotides such as inosine monophosphate (**IMP**), enhances gustatory nerve responses and behavioral preference (Yoshida et al., 2015). Moreover, the presence of *GRM3* and *GRM4* in both oral and gastrointestinal tissues suggests that ducks can sense amino acids both before and after ingestion, contributing to nutrient evaluation and feeding regulation (Yoshida et al., 2015).

Lipid taste is now recognized as a distinct modality, supported by receptors such as GPR120 in birds (Sawamura et al., 2015). In mule ducks, *GPR119* and *GPRC6A* were expressed across all oral tissues studied, with a postprandial upregulation observed in the oral floor mucosa. Functional studies in chickens indicate that fatty acids elicit gustatory nerve responses and influence feeding preferences, suggesting that lipid taste contributes to immediate oral nutrient detection. Beyond sensory perception, although lipids account for less than 3 % of total energy intake in ducks, oral lipid sensing may also prepare digestive and metabolic pathways for lipid absorption (Kawabata et al., 2021).

### Oral floor mucosa: a hotspot for gustatory modulation

Across the taste modalities analyzed, feeding-dependent changes were more pronounced in the oral floor mucosa. This pattern likely reflects species-specific feeding behavior and oral anatomy. Ducks do not chew food extensively; instead, food is retained against the oral floor. Morphological studies have shown that taste buds are abundantly distributed in mandibular and oral floor regions rather than being restricted to the tongue (Stornelli et al., 2000), providing a plausible anatomical basis for the relatively greater responsiveness observed in this tissue.

Feeding appears to alter the transcriptional regulation of taste receptors in bud cells. Postprandial changes in taste receptor gene expression have been reported in several animal models. In rats, nutritional status modulates the expression of peptides and their receptors within taste buds, demonstrating that metabolic condition can influence gene expression in peripheral taste tissues (Chen et al., 2019). In fish, the expression of sweet taste receptors (T1R2 in herbivorous carp) increases after a carbohydrate-rich meal, suggesting that nutrient accumulation in the blood stimulates receptor gene expression involved in detecting nutritional stimuli (Chen et al., 2022). Other study models have also revealed a dynamic response of taste receptors depending on nutritional status, as in trout (Baranek et al., 2024), in chicken (Yoshida et al., 2024). These findings indicate that gustatory signaling is dynamically regulated by nutritional state.

In this postprandial context, nutrient exposure transiently upregulates taste receptor gene expression and associated signaling pathways, reflecting an integration of sensory, metabolic, and hormonal signals that optimizes gustatory responsiveness to the current nutritional state and the composition of ingested food. The binding of sapid molecules to their specific receptors triggers a signaling cascade that transduces the taste message to the brain. Taste perception relies heavily on GPCRs, whose activation initiates intracellular calcium signaling via PLCβ-IP3 pathways (Nelson et al., 2001; Chaudhari and Roper, 2010; Roper, 2013). To investigate this pathway in mule ducks, we measured the expression of two key components of store-operated calcium entry, *STIM1* and *ORAI2*. While their expression showed minor variations across oral tissues and time points, feeding status had no substantial effect, suggesting that the calcium-signaling machinery could be constitutively expressed and ready for activation.

### Neurochemical signaling in oral tissues

Although feeding had no detectable effect on the expression of *STIM1* and *ORAI2*, we examined whether downstream neurochemical signaling was nonetheless modulated postprandially. To this end, we measured levels of 5-HT, a key neurotransmitter released by taste cells following GPCR activation, and its primary metabolite 5-hydroxyindoleacetic acid (5-HIAA), across the three oral tissues. The release of 5-HT activates afferent neurons at the base of taste cells, enabling the transmission of gustatory information through successive neural relays up to the brain (Chaudhari and Roper, 2010). In our experiment, we provided the first evidence in mule ducks of a serotoninergic response to feeding. While 5-HT is well known in mammalian taste buds to rise within minutes postprandially (Roper, 2013), here 5-HT concentrations increased after feeding in all oral tissues with tissue-specific temporal dynamics. Concurrent decreases in 5-HIAA and the 5-HIAA/5-HT ratio indicate reduced serotonin turnover and enhanced gustatory signaling. In mammals, serotonin release from taste bud cells involves calcium-dependent pathways including ERK1/2 signaling, particularly for lipid stimuli (Subramaniam et al., 2016). Our results demonstrate that local serotonergic signaling contributes to oral nutrient detection in birds, with peripheral-central integration remaining to be characterized. Although this effect manifests more rapidly than changes in calcium signaling, it is consistent with findings in mammals, suggesting a link between calcium signaling pathways and serotonin release in taste bud cells of mice and humans (Huang et al., 2005). As previously mentioned, these findings may help explain the observed changes in food-related neuropeptides in the brain, potentially due to impaired activation of the tongue-brain axis.

### Central integration and monoaminergic regulation

After characterizing peripheral responses to feeding, from taste receptor modulation to serotonergic signaling in oral tissues, we investigated whether these signals are transmitted to central regulatory circuits. To this end, we measured hypothalamic neuropeptide expression and monoaminergic metabolism, two key components of central networks controlling appetite, satiety, and feeding motivation in birds (Richards, 2003; Boswell, 2005; Denbow, D. M.; Cogan, T., 2022a). Specifically, we assessed hypothalamic expression of orexigenic (NPY, AgRP) and anorexigenic (POMC, CART) neuropeptides, as well as MC4R, a critical mediator of the melanocortin pathway). In birds, fasting has been reported to increase *NPY* and *AGRP* expression while reducing *POMC* and *CART* levels, although the magnitude and timing of these responses vary across species and experimental conditions, including in ducks (Phillips-Singh et al., 2003; Lees et al., 2017). In our study, all genes were expressed in mule ducks, but none showed significant modulation in response to feeding or over time. This contrasts with the rapid peripheral changes but aligns with previous observations that hypothalamic transcriptional responses are highly variable in avian species (Phillips-Singh et al., 2003). Importantly, neuropeptide activity can also be regulated post-transcriptionally, through mechanisms such as peptide release, neural plasticity, or receptor sensitivity, which are not captured by mRNA measurements (Cone, 2005). Future studies could complement these findings by assessing neuropeptides at the protein level, using Western blotting or immunohistochemistry. In contrast, monoaminergic metabolism was clearly affected by feeding, with enhanced serotonergic and dopaminergic activity. This dissociation between stable hypothalamic neuropeptide expression and dynamic monoaminergic responses suggests that, beyond a purely neuropeptide regulation, feeding may engage circuits related to reward and motivational aspects of food intake. In mammals, such monoaminergic pathways are central to hedonic eating behavior, where sensory perception and palatability can drive food intake independently of homeostatic energy needs (Volkow et al., 2011; Berthoud et al., 2011). Serotonin, in particular, is a key modulator of both homeostatic and hedonic feeding circuits, influencing motivation, reward processing, and feeding-related behaviors. Although hedonic regulation of food intake remains poorly characterized in birds, the feeding-induced modulation of monoaminergic metabolism observed here in mule ducks is consistent with activation of reward-related pathways, suggesting that sensory-driven mechanisms may play a significant role in feeding motivation in this species. This hedonic regulation of food intake, which can dominate the homeostatic control and lead to overconsumption of foods considered appetizing, even when energy requirements have been satisfied (Berthoud et al., 2011), opens new perspectives for the use of palatants to guide ducks toward voluntary hyperphagia.

### Limitations and future perspectives

Several limitations, however, must be acknowledged. First, the relatively short fasting period may not have been sufficient to induce detectable neuropeptide transcriptional changes, whereas longer fasting periods have been shown to elicit strong hypothalamic responses (McConn et al., 2019). Second, most molecular data rely on mRNA expression, which does not always reflect receptor abundance or activity. The current lack of validated, species-specific antibodies for duck taste receptors remains a technical bottleneck for protein-level quantification. In this context, monoamine measurements (5-HT, 5-HIAA, HVA, l-DOPA) provide functional insight. Third, our focus on three oral tissues excludes other chemosensory sites that likely contribute to nutrient detection. In particular, gastrointestinal and hepato-enteric signals are critical regulators of appetite in poultry. Peptides such as ghrelin, cholecystokinin (CCK), glucagon-like peptide 1 (GLP-1), and liver-expressed antimicrobial peptide 2 (LEAP2), together with their receptors (e.g., GHSR), constitute a complex gut-brain axis that finely modulates postprandial responses (Richards, 2003; Denbow, D. M.; Cogan, T., 2022a; b). Future research should focus on the interactions between oral taste receptors, enteric signaling pathways, and olfactory cues to achieve a more integrated understanding of feeding behavior regulation.

Future studies should therefore refine experimental conditions, for example by employing extended fasting protocols to better capture hypothalamic dynamics. Complementing mRNA analyses with protein-level and functional assays would also help assess receptor regulation more directly. Expanding investigations to additional chemosensory sites could uncover further layers of nutrient detection and early satiety signaling. Finally, by identifying key gustatory receptors and neurochemical actors involved in feeding regulation, this work establishes a framework for future nutritional strategies. It now becomes possible to test specific dietary palatants or nutrients and evaluate how their inclusion modulates gustatory and neuroendocrine pathways, ultimately influencing feed intake. Beyond characterizing feeding regulation in young mule ducks, this study raises important questions about developmental plasticity and the timing of sensory maturation. The choice of 4-week-old ducks was based on the functional maturity of the avian gustatory system at this stage, which is already capable of mediating complex oro-sensory responses (Berkhoudt, 1992; Gentle, 1972). Furthermore, this age represents a strategic physiological window in the mule duck’s life cycle, corresponding to the end of the starter feeding period and preceding the major metabolic transitions and intensive fat deposition observed in later development (Chartrin et al., 2007; Bonnefont et al., 2019). The marked postprandial sensitivity observed at 4 weeks may reflect heightened gustatory plasticity, where the system is highly responsive to nutritional stimuli as the animal prepares for the growth phase. Future studies comparing these responses with those of older ducks (e.g., at 10 weeks) could clarify how this sensory-metabolic axis stabilizes as the animal reaches full maturity.

## Conclusion

This study provides the first integrated description of nutrient detection and feeding regulation in young mule ducks, from peripheral gustatory tissues to central monoaminergic pathways. Feeding induced rapid, tissue-specific modulation of taste receptor and signaling gene expression, together with marked changes in serotonergic signaling in the oral cavity, whereas hypothalamic orexigenic and anorexigenic neuropeptides show limited transcriptional responses. In contrast, brain neurotransmitters are clearly regulated by feeding, supporting a key role of serotonergic and dopaminergic systems in the postprandial regulation of feeding. Overall, our work establishes a physiological framework for understanding how oral nutrient detection is translated into central signals controlling feeding in ducks. This foundational knowledge is a necessary prerequisite for future studies aiming to manipulate gustatory inputs or dietary composition in order to modulate feed intake, and ultimately to design nutritional strategies that favor voluntary hyperphagia as an alternative to force-feeding.

Scientific section: Metabolism and Nutrition.

## CRediT authorship contribution statement

**Marie Lasserre:** Writing – review & editing, Writing – original draft, Software, Formal analysis, Data curation. **Yan Fericelli:** Formal analysis, Data curation. **Cécile Heraud:** Software, Formal analysis, Data curation. **Laura-Lou Zwick:** Formal analysis, Data curation. **Karel Sasso:** Formal analysis, Data curation. **Sandra Biasutti:** Formal analysis, Data curation. **Anne Surget:** Formal analysis, Data curation. **Marianne Houssier:** Writing – review & editing, Writing – original draft, Validation, Supervision, Methodology. **Stéphane Panserat:** Writing – review & editing, Writing – original draft, Validation, Supervision, Methodology. **Jérôme Roy:** Writing – review & editing, Writing – original draft, Validation, Supervision, Methodology. **Karine Gontier:** Writing – review & editing, Writing – original draft, Validation, Supervision, Methodology, Funding acquisition, Formal analysis, Data curation, Conceptualization.

## Disclosures

The authors declare the following financial interests/personal relationships which may be considered as potential competing interests:

Karine Gontier reports financial support was provided by Departmental Council of the Landes (CD40). Marie Lasserre reports financial support was provided by University of Pau and Pays de l’Adour (UPPA). Karine Gontier reports financial support was provided by Comité Interprofessionnel des Palmipèdes à Foie Gras (CIFOG). Karine Gontier reports financial support was provided by National Research Institute for Agriculture Food and Environment PHASE Department (API). If there are other authors, they declare that they have no known competing financial interests or personal relationships that could have appeared to influence the work reported in this paper.

## Appendix Supplementary materials

Image, application 1

Image, application 2

Image, application 3

## Footnotes

Supplementary material associated with this article can be found, in the online version, at doi:10.1016/j.psj.2026.107070.
