# Supplementary material for: Postprandial dynamics of sensory receptor and neural mechanisms underlying feeding regulation in mule ducks
Source: Poult Sci. 2026 May 2;105(8):107070. doi: 10.1016/j.psj.2026.107070 (PMC13223829; doi:10.1016/j.psj.2026.107070)
Supplement: Supplementary file 1 [file mmc1.docx]

***Supplementary Table S1. Calibration parameters of UHPLC-FL analysis for indolamines and catecholamines.***

***LC CALIBRATION PARAMETERS***

Calibration parameters of the quantification of analyzed standards obtained with UHPLC-FL system

Abbrev.: abbreviation

t_R_: retention time (min)

LLOD: low limit of detection (fmol)

LLOQ: low limit of quantification (fmol)

R²: goodness of fit

CV: coefficient of variation

|  | **Abbrev.** | **λ_exc_/λ_em_ (nm)** | **t_R_ (min)** | **LLOD (fmol)** | **LLOQ (fmol)** | **Low limit of linearity (pmol)** | **High limit of linearity (pmol)** | **Calibration curve** | **R²** |
| --- | --- | --- | --- | --- | --- | --- | --- | --- | --- |
| ***3,4-Dihydrophenylalanin*** | L-DOPA | 280/330 | 6.252 | 12.35 | 93.56 | 0.28 | 44.99 | y= 6.5E+05 *x - 3.8E+05 | 0.9991 |
| ***5-Hydroxyindolacetic acid*** | 5-HIAA | 285/355 | 13.546 | 24.86 | 138.18 | 0.29 | 45.94 | y= 5.0E+06 *x - 3.3E+06 | 0.9994 |
| ***Serotonin*** | 5-HT | 285/355 | 22.928 | 17.69 | 123.23 | 0.30 | 48.09 | y= 2.4E+06 *x - 1.4E+06 | 0.9994 |
| ***Homovanillic acid*** | HVA | 280/330 | 23.384 | 49.54 | 336.78 | 0.30 | 48.71 | y= 6.7E+04 *x - 3.8E+04 | 0.9996 |
