# Supplementary material for: Postprandial dynamics of sensory receptor and neural mechanisms underlying feeding regulation in mule ducks
Source: Poult Sci. 2026 May 2;105(8):107070. doi: 10.1016/j.psj.2026.107070 (PMC13223829; doi:10.1016/j.psj.2026.107070)
Supplement: Supplementary file 3 [file mmc3.docx]

***Supplementary Figure S1.*** ***First-order kinetic modeling of plasma triglycerides (TG) elimination.***

The elimination rate constant (*k*) was determined by the slope of the linear regression on ln-transformed mean concentrations from the postprandial peak (20 min) to 12 h. Unfed ducks (orange) exhibited a faster clearance rate (*k* = 0.0526 h^-1^) compared to fed ducks (blue, *k* = 0.0255 h^-1^).
